# Change in cardiovascular risk assessment tool and updated Norwegian guidelines for cardiovascular disease in primary prevention increase the population proportion at risk: the Tromsø Study 2015–2016

**DOI:** 10.1136/openhrt-2021-001777

**Published:** 2021-08-30

**Authors:** Amalie Nilsen, Tove Aminda Hanssen, Knut Tore Lappegård, Anne Elise Eggen, Maja-Lisa Løchen, Randi Marie Selmer, Inger Njølstad, Tom Wilsgaard, Laila A Hopstock

**Affiliations:** 1 Department of Community Medicine, UiT The Arctic University of Norway, Tromsø, Norway; 2 Department of Medicine, Nordlands Hospital, Bodo, Norway; 3 Department of Cardiology, University Hospital of North Norway, Tromsø, Norway; 4 Department of Health and Care Sciences, UiT The Arctic University of Norway, Tromsø, Norway; 5 Department of Clinical Medicine, UiT The Arctic University of Norway, Tromsø, Norway; 6 Department of Infectious Disease Epidemiology and Modelling, Norwegian Institute of Public Health, Oslo, Norway

**Keywords:** epidemiology, risk factors, coronary artery disease

## Abstract

**Aims:**

To compare the population proportion at high risk of cardiovascular disease (CVD) using the Norwegian NORRISK 1 that predicts 10-year risk of CVD mortality and the Norwegian national guidelines from 2009, with the updated NORRISK 2 that predicts 10-year risk of both fatal and non-fatal risk of CVD and the Norwegian national guidelines from 2017.

**Methods:**

We included participants from the Norwegian population-based Tromsø Study (2015–2016) aged 40–69 years without a history of CVD (n=16 566). The total proportion eligible for intervention was identified by NORRISK 1 and the 2009 guidelines (serum total cholesterol ≥8 mmol/L, systolic blood pressure ≥160 mm Hg or diastolic blood pressure ≥100 mm Hg) and NORRISK 2 and the 2017 guidelines (serum total cholesterol ≥7 mmol/L, low density lipoprotein (LDL) cholesterol ≥5 mmol/L, systolic blood pressure ≥160 mm Hg or diastolic blood pressure ≥100 mm Hg).

**Results:**

The total proportion at high risk as defined by a risk score was 12.0% using NORRISK 1 and 9.8% using NORRISK 2. When including single risk factors specified by the guidelines, the total proportion eligible for intervention was 15.5% using NORRISK 1 and the 2009 guidelines and 18.9% using NORRISK 2 and the 2017 guidelines. The lowered threshold for total cholesterol and specified cut-off for LDL cholesterol stand for a large proportion of the increase in population at risk.

**Conclusion:**

The population proportion eligible for intervention increased by 3.4 percentage points from 2009 to 2017 using the revised NORRISK 2 score and guidelines.

**Key questions**

**What is already known about this subject?**

- Risk assessment tools and primary prevention guidelines for cardiovascular disease are used to identify individuals eligible for preventive interventions.
- There is a need to balance risk of overtreatment, healthcare cost and potential side effects versus undertreatment.

**What does this study add?**

- We demonstrate how change of cardiovascular risk assessment tool and updated guidelines increase the population proportion eligible for preventive interventions.

**How might this impact on clinical practice?**

- New insights into the impact of risk assessment scoring to identify individuals at risk and accurate estimates of the proportion of the total population at risk are essential for health authorities to target interventions.

## Introduction

Cardiovascular disease (CVD) is a leading cause of death and disability worldwide and an economic burden for the society, thereby calling for an active preventive approach.^1^ Cardiovascular risk prediction tools have been developed to objectively estimate risk and to guide clinical decision-making on initiating, intensifying or discontinuing medical treatment for CVD primary prevention.^2^ The Framingham Risk Score, developed from the Framingham Heart Study in the USA, was the first and most broadly used risk score, and several other risk scores have been developed later.^3^ The European guidelines for CVD primary prevention included the Framingham Risk Score in 1994 and 1998,^4 5^ but studies found the risk score to overestimate risk in European populations.^6 7^ The Systematic Coronary Risk Evaluation (SCORE) risk chart was developed from European cohort studies, and separate risk charts have been developed for low-risk and high-risk regions in Europe.^8^ CVD primary prevention guidelines highlight the use of cardiovascular risk assessment tools to identify high-risk individuals and to indicate when to start treatment, through risk assessment scoring and treatment guidelines for single risk factors.^9 10^ In Norway, the 2009 guidelines for CVD primary prevention^11^ recommended the use of a risk assessment tool to identify high-risk individuals and proposed NORRISK 1, a national calibrated variant of the SCORE prediction model to predict 10-year risk of fatal CVD.^12^ The guideline revision in 2017^13^ recommended the updated risk assessment tool NORRISK 2 to predict 10-year risk of both fatal and non-fatal CVD.^14^

Guideline updates will change the definition of the population at risk. Lowering the threshold for defining individuals at high risk and eligible for primary prevention of CVD causes a larger proportion of individuals in need of lifestyle changes and potentially drug treatment with antihypertensives and/or lipid-lowering drugs. However, a change in threshold can also result in the potential of preventing more fatal and non-fatal events of CVD. There is a need for balancing between the risk of undertreatment with risk of disease or death and overtreatment, medication-related side effects, financial cost and healthcare priorities.^15–17^ The aim of this study was to compare the population proportion at risk and eligibility for intervention as defined by NORRISK 1 and the Norwegian national guidelines from 2009 with NORRISK 2 and the national guidelines from 2017 using a Norwegian population-based sample.

## Methods

### Study population

The Tromsø Study is an ongoing population-based cohort study in the municipality of Tromsø, Northern Norway. The study includes seven surveys conducted between 1974 and 2016 (Tromsø 1–7). Both total birth cohorts and representative samples of the population have been invited, and a total of 45 473 women and men have participated in one or more surveys (attendance 65%–79%).^18^ Data collection includes questionnaires, interviews, biological sampling and clinical examinations. In this study, we included participants from Tromsø 7 (2015–2016), to which all inhabitants aged 40 years or older (n=32 591) were invited, and 21 083 women and men participated (65%). We excluded participants 70 years and older (n=3437), those with previous myocardial infarction (MI) or stroke (n=704) and those without valid data for NORRISK 1 and NORRISK 2 risk calculation (n=376), leaving 16 566 participants for the current analysis. All participants gave written informed consent. The study was approved by the Regional Committee for Medical and Health Research Ethics North (reference 1778/2015).

### Case validation

Cases of MI and stroke were recorded and validated from study entry until 31 December 2014 by the Tromsø Study CVD registry and were available for all participants attending Tromsø 7 and one or more of the previous six surveys. Adjudication of hospitalised and out-of-hospital events was performed by an independent end-point committee reviewing medical records and medical notes, autopsy records and death certificates. The national unique 11-digit identification number allowed linkage to national and local diagnosis registries. Cases of MI and stroke were identified by linkage to the discharge diagnosis registry at the University Hospital of North Norway, the only hospital in the area, with search for International Classification of Diseases described in detail elsewhere.^19^ Due to the lack of validated endpoints after 2014 and among participants attending Tromsø 7 only, we also used self-reported MI or stroke (‘Have you had a heart attack?’ and ‘Have you had a stroke?’) to exclude individuals with previous MI or stroke.

### Measurements

Self-reported data on smoking, diabetes, family history of coronary heart disease (CHD) and use of lipid-lowering and antihypertensive medication were collected via questionnaires. For medication use, a combination of a question (‘Do you use blood pressure lowering drugs?’ and ‘Do you use lipid-lowering drugs?’) and information from a self-reported written list of brand names of regularly used medication (antihypertensives (ATC codes C02, C03, C07, C08 and C09) and lipid-lowering drugs (ATC code C10) was used. Blood pressure was measured on the right arm of all participants (unless in circumstances where this was not possible) three times at 1 min intervals after 2 min seated rest by a Dinamap ProCare 300 monitor (GE Healthcare, Norway), and the mean of the two final readings was used in the analysis. Non-fasting venous blood samples were collected with standard methods, and the samples were analysed within 48 hours for total, LDL and high density lipoprotein (HDL) cholesterol by enzymatic colorimetric methods (with Roche Diagnostics, Mannheim, Germany) and glycated haemoglobin (HbA1c) by high-performance liquid chromatography (with Tosoh G8, Tosoh Bioscience, San Francisco, USA) at the department of laboratory medicine, University Hospital of North Norway. Trained personnel performed all measurements.

### NORRISK 1 and the 2009 guidelines

The multivariable CVD risk assessment tool NORRISK 1 is a Norwegian adaption of the European SCORE model and predicts 10-year risk (%) of death due to atherosclerotic CVD in individuals aged 40–69 years.^12^ Together with the Norwegian guidelines from 2009, NORRISK intended to identify high-risk individuals and guide decision-making in CVD primary prevention. The 10-year risk estimation is based on age, sex, systolic blood pressure, serum total cholesterol and daily smoking habits. Additional risk factors HbA1c levels and first-degree family member with a history of premature CHD were used to recalculate risk with specific cut-offs.^11 12^ Age-specific thresholds are set to determine need of lifestyle advice and/or therapy with antihypertensives and/or lipid-lowering drugs, where indication to initiate treatment is set to NORRISK 1 score: 40–49 years score ≥1%, 50–59 years score ≥5% and 60–69 years score ≥10%. The 2009 guideline defined individuals with elevated values of total cholesterol ≥8 mmol/L, systolic blood pressure ≥160 mm Hg or diastolic blood pressure ≥100 mm Hg to be eligible for intervention regardless of their NORRISK 1 score. In this study, we also calculated the proportion eligible for intervention based on the international definition of hypertension: blood pressure ≥140/90 mm Hg.

### NORRISK 2 and the 2017 guidelines

In 2017, the revised national guidelines for CVD prevention were introduced, and an updated and revised risk assessment tool, NORRISK 2, was presented to identify high-risk individuals eligible for intervention.^13^ NORRISK 2 predicts the 10-year risk (%) of incident MI and stroke combined, including both non-fatal and fatal events of CHD and stroke. The 10-year risk estimation is based on age, sex, systolic blood pressure, serum total cholesterol, daily smoking habits, first-degree family member with a history of premature MI (before the age of 60 years), low serum HDL cholesterol based on sex specific cut-off values (1.0 mmol/L in men and 1.3 mmol/L in women) and use of antihypertensives (where current use increases the score). Selmer *et al*^14^ suggest age-specific thresholds in age groups 45–54, 55–64 and 65–74 years to determine whether an individual is at low, medium or high risk of CVD. Elevated values on single risk factors, that is, serum total cholesterol ≥7 mmol/L, LDL cholesterol ≥5 mmol/L (does not apply for women over 50 years), systolic blood pressure ≥160 mm Hg or diastolic blood pressure ≥100 mm Hg, identify individuals eligible for intervention regardless of the NORRISK 2 score. In addition, in individuals with diabetes, LDL cholesterol ≥2.5 mmol/L and blood pressure ≥140/90 mm Hg indicate eligibility for intervention.^16^ In this study, we also calculated the proportion eligible for intervention by the international definition of hypertension: blood pressure ≥140/90 mm Hg. Additional risk factors (South Asian ethnicity and diagnosis of rheumatoid arthritis) can be used to recalculate the risk score, with specific cut-offs. Abdominal obesity, mental strain and stress are additional risk factors without a specific cut-off value.^13^ In this study, we did not use the proposed additional risk factors to recalculate the NORRISK 2 score.

### Statistics

**Figure 1:**
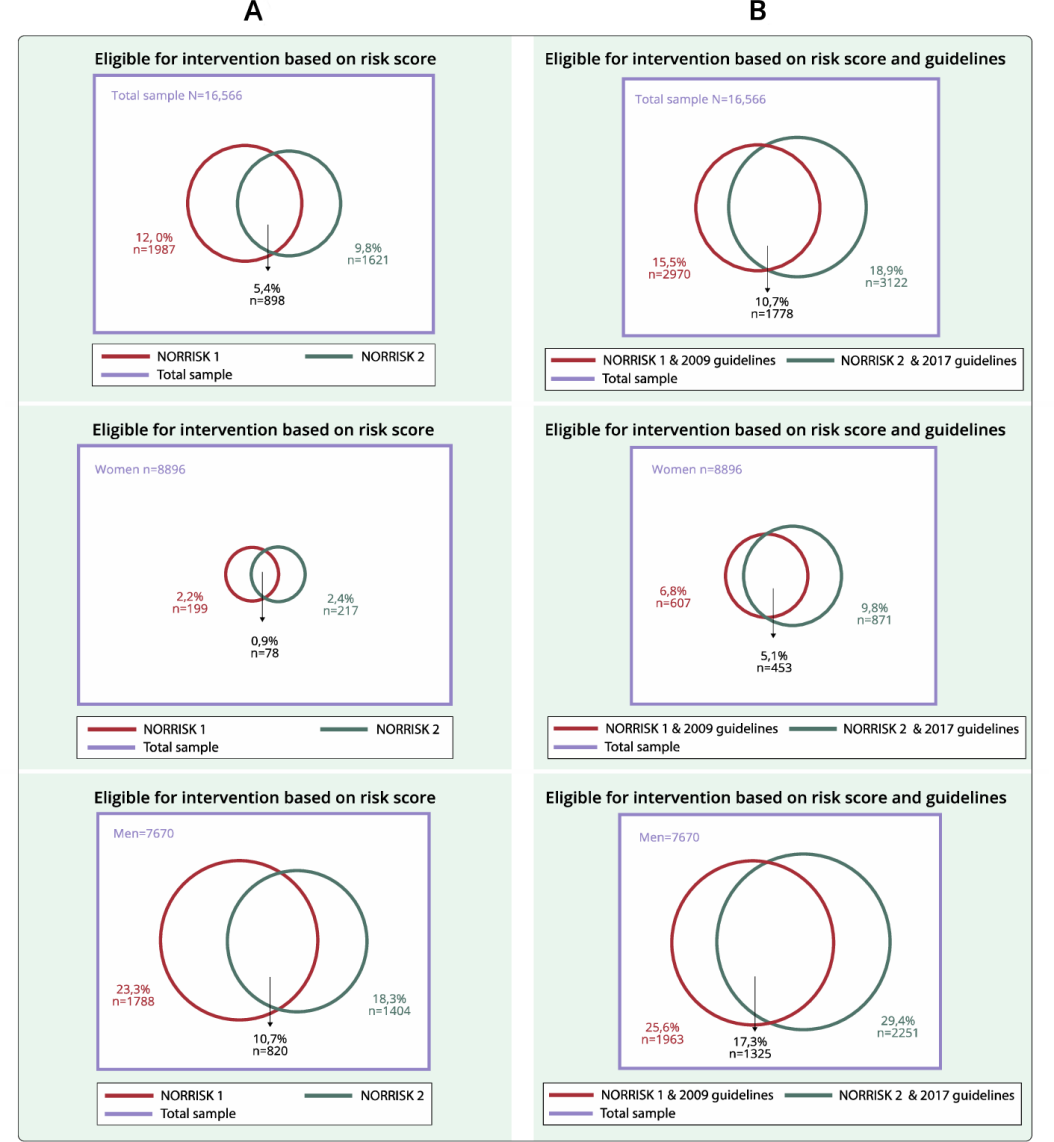
Venn diagram presenting the overlap of identification of high-risk participants defined by NORRISK 1 (red circle) and NORRISK 2 (green circle) in the total sample (purple square) (panel A) and NORRISK 1 and NORRISK 2 combined with single risk factors in 2009 (red circle) and 2017 (green circle) guidelines in the sample (purple square) (panel B) by sex. The Tromsø Study 2015–2016.

We calculated means and proportions of cardiovascular risk factors and sociodemographic factors including self-reported education and cardiovascular risk including measured body mass index (BMI) (normal, overweight and obesity defined as <25, 25–29.9 and 30 kg/m^2^, respectively) and waist circumference (obesity defined as ≥88 and≥102 cm in women and men, respectively) and self-reported current diabetes and physical activity level to present study population characteristics (table 1). We calculated the proportion of participants eligible for intervention according to NORRISK 1 and the 2009 guidelines and NORRISK 2 and the 2017 guidelines (table 2), overall and stratified by sex and age groups. In addition, we calculated the proportion eligible for intervention using NORRISK 1 without the additional risk factors HbA1c level and family history of premature CHD (online supplemental table 1). We also recalculated the proportion in need of intervention with systolic blood pressure ≥140 mm Hg and diastolic blood pressure ≥90 mm Hg as cut-off (online supplemental table 2). To compare sex differences, we used t-tests for continuous variables and χ^2^ tests for categorical variables and McNemar test for pairwise data comparing differences in risk score. Results were considered statistically significant when a p value less than 5% was attained. To visualise the overlap of high-risk participants defined by NORRISK 1 and NORRISK 2 scores, as well as risk score with additional risk factors from the concurrent guidelines, we present area-proportional Venn diagrams (figure 1), overall and by sex. All analyses were performed using Stata V.16 (StataCorp, 2019; Stata Statistical Software).

10.1136/openhrt-2021-001777.supp1 Supplementary data

**Table 1:** Characteristics of study participants by sex and age groups. The Tromsø Study 2015–2016

	Overall n=16 566	Women				Men			
		Overall n=8896	40–49 years n=3286	50–59 years n=3115	60–69 years n=2495	Overall n=7670	40–49 years n=2943	50–59 years n=2579	60–69 years n=2148
Age, years	53.4 (8.4)	53.5 (8.4)	44.5 (2.8)	54.3 (2.9)	64.2 (2.9)	53.4 (8.4)	44.6 (2.8)	54.4 (2.9)	64.3 (2.8)
Total cholesterol, mmol/L	5.5 (1.0)	5.5 (1.0)	5.1 (0.9)	5.7 (1.0)	5.9 (1.0)	5.5 (1.0)	5.5 (1.0)	5.6 (1.0)	5.5 (1.0)
LDL cholesterol, mmol/L	3.6 (1.0)	3.6 (1.0)	3.2 (0.9)	3.7 (0.9)	3.8 (1.0)	3.7 (0.9	3.7 (0.9)	3.9 (0.9)	3.7 (0.9)
HDL cholesterol, mmol/L	1.6 (0.5)	1.7 (0.5)	1.6 (0.4)	1.7 (0.5)	1.8 (0.5)	1.4 (0.4)	1.3 (0.4)	1.4 (0.4)	1.5 (0.4)
Use of lipid-lowering drugs, % (n)	8.5 (1409)	7.9 (706)	1.5 (49)	6.9 (214)	17.8 (443)	9.2 (703)	3.6 (106)	8.5 (219)	17.6 (378)
Systolic blood pressure, mm Hg	126.8 (18.3)	123.3 (18.6)	116.5 (14.6)	123.0 (17.6)	132.6 (20.5)	130.9 (17.1)	126.8 (14.8)	131.0 (17.1)	136.5 (18.3)
Diastolic blood pressure, mm Hg	75.5 (10.1)	72.6 (9.6)	71.2 (9.3)	73.3 (9.7)	73.7 (9.7)	78.9 (9.6)	77.5 (9.4)	79.9 (9.8)	79.7 (9.5)
Use of antihypertensives, % (n)	16.7 (2761)	15.6 (1384)	6.8 (223)	14.5 (451)	28.5 (710)	18.0 (1377)	7.3 (216)	16.0 (412)	34.9 (749)
Current smoking, % (n)	14.7 (2435)	15.3 (1364)	12.7 (417)	18.0 (560)	15.5 (387)	14.0 (1071)	12.7 (373)	15.8 (408)	13.5 (290)
HbA1c, %	5.6 (0.6)	5.6 (0.5)	5.4 (0.5)	5.6 (0.6)	5.7 (0.5)	5.7 (0.6)	5.5 (0.6)	5.7 (0.6)	5.8 (0.7)
Diabetes, % (n)	3.6 (598)	3.1 (279)	2.0 (67)	2.9 (90)	4.9 (122)	4.2 (319)	2.4 (70)	4.2 (108)	6.6 (141)
Body mass index, % (n)									
Normal (<25 kg/m^2^)	33.6 (5548)	42.0 (3729)	44.4 (1457)	42.1 (1310)	38.7 (962)	23.8 (1819)	22.7 (668)	23.5 (606)	25.4 (545)
Overweight (25–29.9 kg/m^2^)	43.1 (7117)	36.2 (3204)	32.6 (1070)	36.7 (1141)	39.9 (993)	51.1 (3913)	50.4 (1479)	51.9 (1337)	51.2 (1097)
Obese (>30 kg/m^2^)	23.4 (3868)	21.9 (1943)	23.0 (753)	21.2 (659)	21.4 (531)	25.1 (1925)	26.9 (790)	24.6 (635)	23.3 (500)
Abdominal obesity*, % (n)	47.6 (7888)	54.0 (4804)	49.0 (1611)	54.6 (1700)	59.8 (1493)	40.2 (3084)	38.3 (1127)	40.4 (1043)	42.6 (914)
Sedentary leisure time physical activity level, %, (n)	13.7 (2232)	12.6 (1097)	13.8 (448)	11.8 (362)	11.9 (287)	15.0 (1135)	16.2 (471)	13.8 (352)	14.8 (312)
Education, % (n)									
Primary	18.3 (3004)	17.7 (1567)	7.3 (240)	17.0 (526)	32.5 (801)	18.9 (1437)	11.8 (347)	19.6 (501)	27.7 (589)
High school	28.1 (4628)	25.9 (2285)	23.4 (766)	27.9 (864)	26.6 (655)	30.8 (2343)	31.6 (926)	32.6 (833)	27.5 (584)
College/university <4 years	20.4 (3360)	19.2 (1691)	21.8 (711)	19.8 (614)	14.8 (366)	21.9 (1669)	22.0 (645)	22.6 (577)	21.0 (447)
College/university ≥4 years	33.2 (5455)	37.2 (3288)	47.5 (1552)	35.3 (1091)	26.2 (645)	28.5 (2167)	34.7 (1017)	25.2 (644)	23.8 (506)

Values are means (SD) and percent (numbers).

*Men ≥102 cm women ≥88 cm.

HbA1c, glycated haemoglobin; HDL, high density lipoprotein; LDL, low density lipoprotein.

**Table 2:** Proportion (%) of participants eligible for intervention defined by NORRISK 1 and the 2009 guidelines and NORRISK 2 and the 2017 guidelines, separate and in combination as total proportion eligible for intervention, by sex and age group. The Tromsø Study 2015–2016

Eligible for intervention	Total n=16 566	Women				Men			
		Overall n=8896	Age group 40–49 n=3286	Age group 50–59 n=3115	Age group 60–69 n=2495	Overall n=7670	Age group 40–49 n=2943	Age group 50–59 n=2579	Age group 60–69 n=2148
**NORRISK 1 high risk, % (n**)	12.0 (1987)	2.2 (199)	0.8 (25)	0.4 (12)	6.5 (162)	23.3 (1788)	26.9 (791)	12.4 (320)	31.5 (677)
**NORRISK 1 low and elevated single risk factors (2009 guidelines**)									
Total cholesterol ≥8 mmol/L, % (n)	1.0 (147)	1.4 (120)	0.4 (13)	1.8 (57)	2.1 (50)	0.5 (27)	0.4 (8)	0.7 (16)	0.2 (3)
Systolic blood pressure ≥160 mm Hg, % (n)	2.7 (397)	3.3 (283)	0.9 (29)	3.2 (98)	6.7 (156)	1.9 (114)	0.5 (10)	3.0 (67)	2.5 (37)
Diastolic blood pressure ≥100 mm Hg, % (n)	0.7 (98)	0.4 (37)	0.3 (11)	0.5 (16)	0.4 (10)	1.0 (61)	0.3 (6)	1.9 (42)	0.9 (13)
**Total proportion eligible for intervention (NORRISK 1 and/or single risk factors), % (n**)	15.5 (2570)	6.8 (607)	2.1 (70)	5.5 (170)	14.7 (367)	25.6 (1963)	27.6 (813)	16.6 (427)	33.7 (723)
**NORRISK 2 high risk, % (n**)	9.8 (1621)	2.4 (217)	0.2 (5)	2.5 (77)	5.4 (135)	18.3 (1404)	7.5 (221)	23.5 (605)	26.9 (578)
**NORRISK 2 low and elevated single risk factors (2017 guidelines**)									
Total cholesterol ≥7 mmol/L*, % (n)	2.8 (414)	1.2 (100)	3.1 (100)	*	*	5.0 (314)	5.8 (159)	5.0 (99)	3.6 (56)
LDL cholesterol ≥5 mmol/L*, % (n)	3.6 (535)	1.3 (112)	3.4 (112)	*	*	6.8 (423)	7.3 (198)	7.2 (143)	5.2 (82)
Systolic blood pressure ≥160 mm Hg, % (n)	3.1 (470)	3.5 (302)	1.0 (33)	2.8 (85)	7.8 (184)	2.7 (168)	1.8 (48)	2.3 (46)	4.7 (74)
Diastolic blood pressure ≥100 mm Hg, % (n)	0.6 (95)	0.4 (34)	0.4 (14)	0.4 (11)	0.4 (9)	1.0 (61)	0.9 (24)	0.9 (18)	1.2 (19)
Diabetes and LDL cholesterol >2.5 mmol/L, % (n)	2.7 (399)	2.5 (213)	1.7 (54)	2.3 (70)	3.8 (89)	3.0 (186)	1.8 (50)	3.2 (63)	4.7 (73)
Diabetes and blood pressure ≥140/90 mm Hg, % (n)	0.9 (140)	0.9 (76)	0.3 (10)	0.8 (24)	1.8 (42)	1.0 (64)	0.4 (10)	0.8 (15)	2.5 (39)
**Total proportion eligible for intervention (NORRISK 2 and/or single risk factors), % (n**)	18.9 (3122)	9.8 (871)	6.9 (227)	7.5 (233)	16.5 (411)	29.4 (2251)	18.6 (548)	34.2 (881)	38.3 (822)

Values are percentages (numbers).

*Indication to start intervention at total cholesterol concentration ≥7 mmol/L and LDL cholesterol ≥5 mmol/L does not apply for women >50 years.

LDL, low density lipoprotein.

## Results

### Study population and CVD risk factors

Study population characteristics are presented in table 1. Mean age was 53 years for both sexes. Compared with women, men had higher LDL cholesterol, blood pressure, prevalence of obesity (BMI >30 kg/m^2^), diabetes, sedentary lifestyle and use of lipid-lowering drugs and antihypertensives but lower HDL cholesterol, prevalence of smoking, abdominal obesity and a lower proportion with higher education.

### NORRISK 1 versus NORRISK 2

The total proportion at high risk (ie, eligible for intervention) defined by risk score only was 12.0% for NORRISK 1 and 9.8% for NORRISK 2 (table 2). The proportion of high-risk individuals using NORRISK 1 was 8.6% calculated without the additional risk factors HbA1c and family history (online supplemental table 1). In all age groups, a higher proportion of men than women was defined as high-risk individuals (p<0.001) (table 2). Among men aged 40–49 years, a larger proportion was identified as high risk using NORRISK 1 compared with NORRISK 2 (p<0.001), whereas in men aged 50–59 years, more men were identified as high risk using NORRISK 2 (p<0.001).

### Total proportion eligible for intervention

The total proportion eligible for intervention identified by risk score or elevated values for single CVD risk factors was 3.4 percentage points higher using NORRISK 2 and the 2017 guidelines compared with NORRISK 1 and the 2009 guidelines (18.9% vs 15.5%). The total proportion eligible for intervention was higher using NORRISK 2 and the 2017 guidelines in both sexes and all age groups, except among men aged 40–49 years (table 2). In women, the proportion eligible for intervention increased by 3.0 percentage points from 6.8% to 9.8%, and the increase among men was 3.8 percentage points from 25.6% to 29.4% by NORRISK 1 and the 2009 guidelines, compared with NORRISK 2 and the 2017 guidelines, respectively. Overall, participants defined as being at low risk by risk score were to a greater extent identified as eligible for intervention by single risk factors when using the 2017 guidelines compared with the 2009 guidelines. This was due to change in the cut-off value for serum total cholesterol and the introduction of a specified value for LDL cholesterol. One percent of the participants with low risk by NORRISK 1 had total cholesterol above the threshold of ≥8 mmol/L, whereas the lowering of the threshold in the 2017 guideline to ≥7 mmol/L increased the proportion to 2.8% in individuals with low risk by NORRISK 2. Specifying a threshold for LDL cholesterol to ≥5 mmol/L in the 2017 guideline identified 3.6% individuals above threshold among individuals identified as low risk by NORRISK 2. Among participants defined as being at low risk by NORRISK 1, systolic blood pressure identified an additional 2.7% of the study population as high risk with the 2009 guideline, and 3.1% of participants defined as low risk by NORRISK 2 were identified as being at high risk by the 2017 guidelines. When including the diabetes-specific threshold in the 2017 guidelines for those with self-reported diabetes, 2.7% had LDL cholesterol ≥2.5 mmol/L, and 0.9% had blood pressure ≥140/90 mm Hg but were defined as low risk by NORRISK 2. A larger proportion of women compared with men was identified as eligible for intervention by single risk factors only using the 2009 guidelines, while applying single risk factors only to the 2017 guidelines identified a higher proportion of men than women eligible for intervention. When we recalculated the total proportion eligible for intervention based on systolic blood pressure ≥140 mm Hg and diastolic blood pressure ≥90 mm Hg, we found 29.3% based on NORRISK 1 and the 2009 guidelines and 32.4% using NORRISK 2 and the 2017 guidelines (online supplemental table 2).

### Overlap between risk scores only and risk scores and the guidelines combined

Among individuals identified as high risk by risk score only, NORRISK 1 identified in total 12.0% (2.2% of women and 23.3% of men) as high risk, while NORRISK 2 identified in total 9.8% (2.4% of women and 18.3% of men) as high risk. The overlapping proportion identified as high risk in both risk scores was in total 5.4% (0.9% of women and 10.7% of men). Combining NORRISK 1 and the 2009 guidelines, 15.5% in total (6.8% women and 25.6% men) was identified as eligible for intervention, while when using NORRISK 2 and the 2017 guidelines, the proportion was 18.9% in total (9.8% women and 29.4% men). Overall, the overlapping proportion of 10.7% (5.1% women and 17.3% men) was identified as eligible for intervention in both risk scores with their respective guidelines (figure 1).

## Discussion

In this study, we compared the proportion at high CVD risk and eligible for intervention using two consecutive versions of guidelines and risk assessment tools in a Norwegian general population of women and men aged 40–69 years. The main finding is that the proportion eligible for intervention increased from 15.5% using the risk assessment tool NORRISK 1 and the 2009 guidelines to 18.9% using the revised NORRISK 2 and the 2017 guidelines.

### Change in cardiovascular risk assessment tool

The proportion of high-risk individuals defined by risk score only was lower using the updated NORRISK 2 compared with the previous NORRISK 1. This can be explained by the fundamental differences in the risk scores, as they measure different endpoints and thus are not directly comparable. NORRISK 1 predicts 10-year risk of fatal CVD, whereas NORRISK 2 predicts the 10-year risk of MI, stroke and fatal CVD.^12 14^ The European guidelines for CVD primary prevention encourage the calibration of risk assessment tools to the target population by adjusting for secular changes in risk factor levels and CVD mortality.^20^ A reduction over time in the major CVD risk factors serum total cholesterol, blood pressure and smoking in the general population has been shown both in large international studies^21 22^ and in the Tromsø Study population,^19 23 24^ and we have previously demonstrated a decline in total CVD risk in the Tromsø Study^25^ similar to findings from the UK^26^ and the USA.^27^ Further, there has been a major decline in mortality and morbidity of CVD in Norway.^28^ The reduction in risk factors, morbidity and mortality over time can explain the lower proportion eligible for intervention by the updated risk assessment tool NORRISK 2. NORRISK 1 is a national calibrated version of the European SCORE algorithm, based on national mortality rates from 1993 to 2003, and mean level risk factors from Norwegian Health Surveys from 2000 to 2003,^12^ while NORRISK 2 is based on the 10-year follow-up of a large population-based cohort (Cohort of Norway (CONOR)) through linkage to the Cardiovascular Disease in Norway (CVDNOR) project, a database of CVD hospital discharge diagnoses and mortality in Norway in 1994–2009.^14^ Another explanation of this finding can be the use of additional risk factors in our analysis, where we included additional risk factors in the calculation of NORRISK 1 (HbA1c levels and family history of CHD) and did not include the additional risk factors (rheumatoid arthritis, South Asian ethnicity, abdominal obesity and/or mental stress) in the calculation of NORRISK 2. A recent study found NORRISK 2 to underestimate CVD risk in South Asians and proposed an update (NORRISK 2-SADia) improving the predictions of 10-year risk in this population.^29^ In our study, valid data regarding the proposed additional risk factors with specified cut-offs (ethnicity and diagnosis of rheumatoid arteritis) were not available.^30^ Almost half of the study population had abdominal obesity, and in real patient consultations, this could lead to a higher proportion at high risk using NORRISK 2. However, this risk factor is without a multiplication factor and hence not used to calculate the proportion eligible for intervention in this study.

### Single risk factors defined in treatment guidelines

In this study, we found that the updated risk score with additional guidelines increased the proportion of participants eligible for intervention, where the decrease in threshold for total cholesterol levels and a defined value of LDL cholesterol stand for a large proportion of this increase. The impact on the risk of CVD by lowering cholesterol levels is well known. A lowering of LDL cholesterol levels by 1 mmol/L corresponds to a 20%–25% reduction in non-fatal MI and death due to CVD.^10^ Our findings are in line with a study from Denmark where the authors found that the updated 2019 European Society of Cardiology/European Atherosclerosis Society (ESC/EAS) guidelines doubled the proportion of individuals eligible for statin therapy compared with the previous guidelines,^31^ findings that are similar to other studies.^32 33^

For blood pressure, there was no difference in threshold between the 2009 and 2017 guidelines where systolic blood pressure ≥160 mm Hg and diastolic blood pressure ≥100 mm Hg urge immediate start of pharmacological treatment (regardless of NORRISK score), in line with the European ESC/EAS guidelines.^34^ In the American College of Cardiology/American Heart Association (ACC/AHA) guidelines, the recommendation is that blood pressure ≥140/90 mm Hg should lead to direct initiation of antihypertensive drugs.^35^ Hypertension is defined as blood pressure ≥140/90 mm Hg; however, in Norway, lifestyle modification is encouraged before starting medical treatment. By replacement of blood pressure cut-off from ≥160/100 mm Hg to ≥140/90 mm Hg, we found that the proportion eligible for intervention by NORRISK 1 and the 2009 guidelines increased by 13.8 percentage points, and when using NORRISK 2 and the 2017 guidelines, the proportion eligible for intervention increased by 13.4 percentage points. However, in the important debate regarding treatment target in blood pressure levels in primary prevention, it has been suggested that lifestyle modification should be emphasised to a larger degree before initiating pharmacological treatment.^36^

### Combining risk score and additional risk factors

To the best of our knowledge, there have been few previous studies combining both risk score assessment tools and additional guidelines to compare the proportion at risk of CVD and eligibility of intervention in a general population. A study from Germany used the risk assessment tool SCORE Deutschland with additional risk factors (diabetes, total cholesterol ≥8 mmol/L, renal insufficiency and stage 3 hypertension (blood pressure ≥180/110 mm Hg)) and found 13.4% of the study population to be at high risk of 10-year CVD mortality.^37^ Interestingly, the authors found that among men, the majority of high-risk individuals were eligible for intervention because of SCORE ≥5%, contrary to women where the majority of women were classified as high risk based on additional risk factors,^37^ which is in line with our findings. Other studies have also found that adding comorbidities and single risk factors increases the proportion of individuals at high risk, demonstrating the challenge of comparing our findings with other studies.^38 39^

In conclusion, we found that updated CVD primary prevention guidelines increased the proportion at risk and eligible for intervention by 3.4 percentage points in individuals aged 40–69 years, where the increase was 3.0 percentage points in women and 3.8 percentage points in men. In Norway, there are about 2.1 million inhabitants aged 40–69 years.^40^ Therefore, this increase causes almost 70 000 more individuals eligible for intervention using NORRISK 2 and the 2017 guidelines in this age group. Individuals identified to be at high risk and eligible for intervention may be given the opportunity from their primary physician to make necessary lifestyle changes. The guideline^13^ suggests that individuals at high risk are given 3–12 months to make changes such as smoking cessation, increased physical activity and dietary changes to lower blood pressure and cholesterol levels before considering initiating drug treatment with antihypertensives and/or lipid-lowering drugs. However, among individuals with very high blood pressure, cholesterol levels or high total risk, drug treatment may be initiated directly. An increase of 3.4 percentage points means a higher number of individuals in need of time from their primary physician to give lifestyle advice, follow up the effect of this advice and assess whether to start drug treatment. Among individuals that start drug treatment, there is a need for follow-up to evaluate drug efficacy and whether treatment targets are achieved, as well as side effects. Change in the guidelines of CVD prevention may lead to a higher burden of the healthcare system, but this also translates into a higher number of individuals who can avoid a fatal or non-fatal event of CVD. The main goal in the use of risk assessment tools is to identify the right individuals to keep the balance between avoiding the potential negative effects such as side effects, overtreatment, undertreatment and a higher cost for the healthcare system on one side and preventing high-risk individuals from developing CVD on the other.^2^

## Conclusion

The population proportion eligible for intervention increased by 3.4 percentage points from 2009 to 2017 using the revised NORRISK 2 score and guidelines, where the lowering of threshold in total cholesterol and specified cut-off for LDL cholesterol stand for a large proportion of the increase in population at risk.

### Strengths and limitations

A strength of this study is the use of a sample from a large population-based study, with validated endpoints for exclusion of prevalent cases and risk factor measurements performed by trained personnel using standardised protocols and instruments. A limitation is that participants in population-based studies in general tend to be healthier than non-attenders. This potential selection bias might cause underestimation of the true population proportion in need of intervention.

## Data Availability

Data may be obtained from a third party and are not publicly available.
