# Supplementary material for: Change in cardiovascular risk assessment tool and updated Norwegian guidelines for cardiovascular disease in primary prevention increase the population proportion at risk: the Tromsø Study 2015–2016
Source: Open Heart. 2021 Aug 30;8(2):e001777. doi: 10.1136/openhrt-2021-001777 (PMC8407203; doi:10.1136/openhrt-2021-001777)
Supplement: Supplementary data [file openhrt-2021-001777supp001.pdf]

Supplementary table 1: Proportion of participants in eligible for intervention defined by NORRISK 1 without additional risk factors HbA1c and first-degree family member with premature CHD and 2009 guidelines, NORRISK 2, 2017 guidelines, separate and in combination as total proportion eligible for intervention, by sex and age group. The Tromsø Study 2015-2016

**Eligible for intervention**		**Women**				**Men**			
	**Total** N=16566	**Overall** n=8896	**Age group 40-49** n=3286	**Age group 50-59** n=3115	**Age group 60-69** n=2495	**Overall** n=7670	**Age group 40-49** n=2943	**Age group 50-59** n=2579	**Age group 60-69** n=2148
**NORRISK 1 high risk, % (n)**	8.6 (1421)	1.2 (104)	0.2 (6)	0.1 (3)	3.8 (95)	17.2 (1317)	20.7 (608)	7.4 (191)	24.1 (518)
**NORRISK 1 low & elevated single risk factors (2009 guidelines)**									
Total cholesterol ≥8 mmol/L, % (n)	1.1 (166)	1.5 (129)	0.4 (13)	1.9 (59)	2.4 (57)	0.6 (37)	0.4 (9)	1.0 (23)	0.3 (5)
Systolic blood pressure ≥160 mm Hg, % (n)	3.1 (464)	3.5 (309)	1.0 (32)	3.6 (101)	7.3 (176)	2.4 (155)	0.6 (15)	3.7 (89)	3.1 (51)
Diastolic blood pressure ≥100 mm Hg, % (n)	0.8 (115)	0.5 (42)	0.4 (13)	0.6 (18)	0.5 (11)	1.2 (73)	0.3 (7)	2.1 (50)	1.0 (16)
**Total proportion eligible for intervention (NORRISK 1 and/or single risk factors), % (n)**	12.6 (2091)	6.1 (545)	1.6 (54)	5.3 (166)	13.0 (325)	20.2 (1546)	21.6 (636)	12.8 (330)	27.0 (580)
**NORRISK 2 high risk, & (n)**	9.8 (1621)	2.4 (217)	0.2 (5)	2.5 (77)	5.4 (135)	18.3 (1404)	7.5 (221)	23.5 (605)	26.9 (578)
**NORRISK 2 low & elevated single risk factors (2017 guidelines)**									
Total cholesterol ≥7 mmol/l *, % (n)	2.8 (414)	1.2 (100)	3.1 (100)	*	*	5.0 (314)	5.8 (159)	5.0 (99)	3.6 (56)
LDL cholesterol ≥5 mmol/L *, % (n)	3.6 (535)	1.3 (112)	3.4 (112)	*	*	6.8 (423)	7.3 (198)	7.2 (143)	5.2 (82)
Systolic blood pressure ≥160 mm Hg, % (n)	3.1 (470)	3.5 (302)	1.0 (33)	2.8 (85)	7.8 (184)	2.7 (168)	1.8 (48)	2.3 (46)	4.7 (74)
Diastolic blood pressure ≥100 mm Hg, % (n)	0.6 (95)	0.4 (34)	0.4 (14)	0.4 (11)	0.4 (9)	1.0 (61)	0.9 (24)	0.9 (18)	1.2 (19)
Diabetes & LDL cholesterol >2.5 mmol/L, % (n)	2.7 (399)	2.5 (213)	1.7 (54)	2.3 (70)	3.8 (89)	3.0 (186)	1.8 (50)	3.2 (63)	4.7 (73)
Diabetes & blood pressure ≥140/90 mm Hg, % (n)	0.9 (140)	0.9 (76)	0.3 (10)	0.8 (24)	1.8 (42)	1.0 (64)	0.4 (10)	0.8 (15)	2.5 (39)
**Total proportion eligible for intervention (NORRISK 2 and/or single risk factors), % (n)**	18.9 (3122)	9.8 (871)	6.9 (227)	7.5 (233)	16.5 (411)	29.4 (2251)	18.6 (548)	34.2 (881)	38.3 (822)

Values are percentages (numbers).
*Indication to start intervention at total cholesterol concentration ≥7 mmol/L and LDL-cholesterol ≥ 5 mmol/L does not apply for women >50 years.

Supplementary table 2: Proportion of individuals eligible for intervention defined by NORRISK 1, 2009 guidelines, NORRISK 2, 2017 guidelines with blood pressure cut off 140/90 mmHg, separate and in combination as total proportion eligible for intervention, by sex and age group. The Tromsø Study 2015-2016.

**Eligible for intervention**		**Women**				**Men**			
	**Total N=16566**	**Overall n=8896**	**Age group 40-49 n=3286**	**Age group 50-59 n=3115**	**Age group 60-69 n=2495**	**Overall n=7670**	**Age group 40-49 n=2943**	**Age group 50-59 n=2579**	**Age group 60-69 n=2148**
**NORRISK 1 high risk, % (n)**	12.0 (1987)	2.2 (199)	0.8 (25)	0.4 (12)	6.5 (162)	23.3 (1788)	26.9 (791)	12.4 (320)	31.5 (677)
**NORRISK 1 low & elevated single risk factors (2009 guidelines)**									
Total cholesterol ≥8 mmol/L, % (n)	1.0 (147)	1.4 (120)	0.4 (13)	1.8 (57)	2.1 (50)	0.5 (27)	0.4 (8)	0.7 (16)	0.2 (3)
Systolic blood pressure ≥140 mm Hg, % (n)	17.4 (2534)	16.7 (1455)	6.9 (225)	16.7 (517)	30.6 (713)	18.3 (1079)	9.4 (202)	22.4 (506)	25.2 (371)
Diastolic blood pressure ≥90 mm Hg, % (n)	6.0 (870)	4.2 (366)	3.3 (107)	5.2 (161)	4.2 (98)	8.6 (504)	5.2 (111)	12.3 (278)	7.8 (115)
**Total proportion eligible for intervention (NORRISK 1 and/or single risk factors)**	29.3 (4845)	20.5 (1822)	9.1 (298)	19.6 (609)	36.7 (915)	39.4 (3023)	35.4 (1043)	35.1 (904)	50.1 (1076)
**NORRISK 2 high risk, % (n)**	9.8 (1621)	2.4 (217)	0.2 (5)	2.5 (77)	5.4 (135)	18.3 (1404	7.5 (221)	23.5 (605)	26.9 (578)
**NORRISK 2 low & elevated single risk factors (2017 guidelines)**									
Total cholesterol ≥7 mmol/l *, % (n)	2.8 (414)	1.2 (100)	3.1 (100)	*	*	5.0 (314)	5.8 (159)	5.0 (99)	3.6 (56)
LDL cholesterol ≥5 mmol/L *, % (n)	3.6 (535)	1.3 (112)	3.4 (112)	*	*	6.8 (423)	7.3 (198)	7.2 (143)	5.2 (82)
Systolic blood pressure ≥140 mm Hg, % (n)	18.4 (2752)	16.9 (1468)	7.2(235)	15.8 (481)	31.9 (752)	20.5 (1284)	15.4 (420)	19.6 (386)	30.5 (478)
Diastolic blood pressure ≥90 mm Hg, % (n)	6.1 (914)	4.3 (371)	3.5 (116)	4.8 (146)	4.6 (109)	8.7 (543)	8.1 (219)	10.0(197)	8.1 (127)
Diabetes & LDL cholesterol >2.5 mmol/L, % (n)	2.7 (399)	2.5 (213)	1.7 (54)	2.3 (70)	3.8 (89)	3.0 (186)	1.8 (50)	3.2 (63)	4.7 (73)
Diabetes & blood pressure ≥140/90 mm Hg, % (n)	0.9 (140)	0.9 (76)	0.3 (10)	0.8 (24)	1.8 (42)	1.0 (64)	0.4 (10)	0.8 (15)	2.5 (39)
**Total proportion eligible for intervention (NORRISK 2 and/or single risk factors)**	32.4 (5360)	22.8 (2029)	13.4 (439)	20.6 (641)	38.0(949)	43.3 (3331)	31.6 (930)	47.1 (1214)	55.3(1187)

Values are percentages (numbers).
* Indication to start intervention at total cholesterol concentration ≥7 mmol/L and LDL-cholesterol ≥ 5 mmol/L does not apply for women >50 years.
